# High prevalence of hyperkalemia in Brazilian chronic dialysis patients and differences across geographic regions

**DOI:** 10.1590/2175-8239-JBN-2022-0053en

**Published:** 2022-07-29

**Authors:** Fabiana Baggio Nerbass, Helbert do Nascimento Lima, Ricardo Sesso, Jocemir Ronaldo Lugon

**Affiliations:** 1 Fundação Pró-Rim, Joinville, SC, Brazil; 2 Universidade da Região de Joinville, Joinville, SC, Brazil; 3 Universidade Federal de São Paulo, São Paulo, SP, Brazil; 4 Universidade Federal Fluminense, Niterói, RJ, Brazil

**Keywords:** Dialysis, Serum Potassium, Hyperkalemia, Epidemiology, Diálise, Potássio Sérico, Hiperpotassemia, Epidemiologia

## Abstract

**Introduction:**

Hyperkalemia is a common multifactorial condition of people on chronic dialysis and is associated with mortality. We aimed to inform and discuss the prevalence of hyperkalemia in a large population of chronic dialysis patients in Brazil and its geographic regions.

**Methods:**

Prevalence of hyperkalemia (serum potassium ≥6.0 mEq/L) was assessed in the Brazilian Dialysis Survey (BDS) in July 2019, an online survey of voluntary participation in which all dialysis centers registered at the Brazilian Society of Nephrology were invited.

**Results:**

Approximately one-third (n=263 of 805) of the Brazilian dialysis clinics participated. The prevalence of hyperkalemia in the whole population was 16.1% (n=7,457 of 46,193; 95%CI=15.8-16.5%,), and varied from 12.1% in the North to 18.7% in the Northeast.

**Conclusion:**

We found a high prevalence of hyperkalemia in a large Brazilian chronic dialysis population. A nationwide investigation of risk factors, treatment options, and whether this high prevalence contributes to dialysis mortality is warranted.

## Resumo

**Introdução:**

A hipercalemia é uma condição multifatorial comum em pessoas em diálise crônica e está associada à mortalidade. Nosso objetivo foi informar e discutir a prevalência de hipercalemia em uma grande população de pacientes em diálise crônica no Brasil e diferenças entre as regiões geográficas.

**Métodos:**

A prevalência de hipercalemia (potássio sérico ≥6,0 mEq/L) foi avaliada por meio do Censo Brasileiro de Diálise (CBD) em Julho de 2019, uma pesquisa online de participação voluntária na qual foram convidados todos os centros de diálise registrados na Sociedade Brasileira de Nefrologia.

**Resultados:**

Aproximadamente um terço (n=263 de 805) das clínicas de diálise brasileiras participaram. A prevalência de hipercalemia na população total foi de 16,1% (n=7.457 de 46.193; IC95%=15,8-16,5%), e variou de 12,1% no Norte a 18,7% no Nordeste.

**Conclusão:**

Encontramos uma elevada prevalência de hipercalemia em umagrande população brasileira em diálise crônica. É necessária uma investigação nacional dos fatores de risco, opções de tratamento e se esta alta prevalência contribui para a mortalidade desta população.

## Introduction

High serum potassium is a common condition recognized as a risk factor for sudden death and all-cause mortality in the dialysis population.^1,2^ Serum potassium balance is influenced by several factors, including dietary potassium intake (quantity and bioavailability), dialysis parameters (dialysate potassium, bicarbonate, and glucose concentration), medications (β-blockers, heparin, renin-angiotensin system inhibitors (RAAS)), and other conditions (acidosis, insulin deficiency, hyperosmolality).^3^

Geographic location and climate parameters can also influence serum potassium levels. In a previous study, the seasonal variability was assessed in a center in an area of tropical-savannah-like climate in Brazil when the highest levels were reported in autumn and the lowest in spring.^4^ Seasonal variations were also reported in studies performed in other countries.^5-8^ Regarding geographic location, a large study from the USA found that patients from the Mediterranean-type climate had higher mean serum potassium than those from areas with continental and subtropical climates.^5^

Brazil has a vast territory with significant variations in climate and cultural habits, and investigations regarding the overall prevalence and distribution of hyperkalemia from a nationwide survey are lacking.

In 2019, the Brazilian Dialysis Survey (BDS), the main source of clinical and epidemiological data on chronic dialysis in our country, included a question about predialysis hyperkalemia status for the first time. Thus, in this brief communication, we aimed to inform and discuss the prevalence of hyperkalemia in chronic dialysis patients in Brazil and its geographic regions.

## Methods

### Data collection and analysis

Dialysis clinics filled out an online questionnaire (BDS) available on the Brazilian Society of Nephrology (BSN) website. It contained questions about sociodemographic, clinical, and therapeutic variables of patients on chronic dialysis (hemodialysis or peritoneal dialysis). The data for each center were grouped rather than reported individually. To calculate hyperkalemia prevalence (and 95% confidence intervals (95%CI)), we used the informed total number of active patients in the center and those with serum potassium ≥6.0 mEq/L in their routine laboratory analysis in July 2019. Data collection was performed before the start of a session for hemodialysis patients and during their monthly visit for peritoneal dialysis patients. Participation in the survey was voluntary, and all dialysis centers registered at BSN were invited to participate by email and media. After the initial invitation, additional strategies were used to increase participation, such as monthly reminders emailed to centers.

## Results

The total response rate of the survey was 39% (314 out of 805 centers). Thirty-three percent (n=263) of the centers answered the question about the number of patients with serum potassium above or equal to 6 mEq/L, totalizing data from 46,193 patients. Across the five regions, the lowest response rate was from the Mid-West (n=19 of 76; 25%), followed by the North (n=15 of 46; 32.6%), Northeast (n=49 of 149; 32.9%), Southeast (n=125 of 377; 33.2%), and South (n=55 of 157; 35%) regions. Concerning dialysis modality, 92.7% were on hemodialysis and 7.3% on peritoneal dialysis.

**Figure 1:**
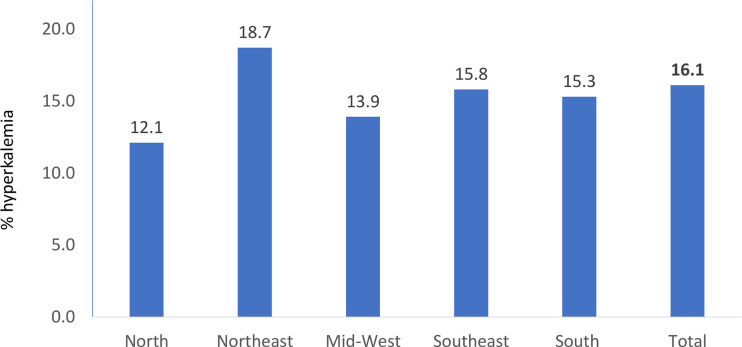
Prevalence of hyperkalemia in the whole population and across geographic regions.

The prevalence of hyperkalemia in the whole population was 16.1% (n=7,457 of 46,193; 95%CI= 15.8-16.5%), and the distribution across regions is shown in Figure 1. The North region had the lowest prevalence (n=309 of 2,545; 12.1%, 95%CI= 10.9-13.5%), followed by the Mid-West (n=435 of 3,140; 13.9%, 95%CI= 12.7-15.1%), the South (n=1,049 of 6,875; 15.3%, 95%CI= 14.4-16.1%), the Southeast (n=3,491of 22,037; 15.8%, 95%CI= 15.4-16.3%), and the Northeast (n=2,173 of 11,596; 18.7%, 95%CI= 18-19.5%) regions.

## Discussion

In this analysis, which included approximately one-third of all chronic dialysis patients (n=46,193) in Brazil in July 2019, the prevalence of hyperkalemia was 16.1% and varied across the five geographic regions.

Our overall prevalence was higher than in other populational studies with a similar cutoff for hyperkalemia (≥ or >6 mEq/L) in hemodialysis patients. In the Dialysis Outcomes and Practice Patterns Study (DOPPS) that included 55,183 patients from 20 countries in 2017, 8% had hyperkalemia,^9^ while in a large American sample with 74,219 participants in 2007, the prevalence was 4.5%.^2^ Other results from smaller studies showed a 7.8 and 13% prevalence in a Spanish^10^ and Greek^8^ population, respectively.

As stated before, serum potassium is influenced by several modifiable factors. Since we did not collect information regarding these factors in this survey, it is impossible to compare clinical practices with international data.

The lack of control of metabolic acidosis is one of the features that may influence the high hyperkalemia prevalence. Since 1996, the determination of bicarbonate levels has not been included as a compulsory routine laboratory test in the reimbursement package for public hemodialysis patients, making it difficult to detect this often silent condition and implement a treatment when needed.^11^

Another possible cause is the wide use of hyperkalemic medications. In a Brazilian multicenter study with 195 hemodialysis participants, 53% used beta-blockers and 45% RAAS.^12^

Changes in dialysis prescriptions by extending dialysis sessions or increasing their frequency, may be difficult to implement due to logistics and public healthcare reimbursement restrictions. It can also affect adequate control of potassium levels.

Regarding dietetic influence, large national epidemiological data and studies performed with Brazilian populations in hemodialysis found a low overall consumption of dietary potassium.^13,14^ Moreover, other studies have demonstrated a lack of association between dietary and serum potassium in people on chronic dialysis .^15,16^

The prevalence of hyperkalemia was high in all five regions and ranged from 12.1% in the North to 18.7% in the Northeast, corresponding to a 54% variation. Unfortunately, the lack of data regarding individual characteristics, dialysis and drug prescriptions, and dietary habits prevent us from investigating possible reasons for the observed differences. The association between dietary and serum potassium has not been extensively studied in Brazilian dialysis patients. There is a marked difference in dietary habits across regions, which could impact potassium levels of dialysis patients.

The results of the DOPPS study showed that serum potassium above 6.0 mEq/L increased arrhythmia by 21% and all-cause mortality by 12 to 33%.^9,17^ Our results should raise the awareness of the nephrology community to this common life-threatening condition.

We highlight as limitations the electronic data collection by voluntary completion, the grouping of patient data by dialysis center, and the lack of validation of responses. Also, the data were obtained in only one month of the year (July). Therefore, a longer study period is needed to better evaluate the prevalence of hyperkalemia and regional variations. Finally, we could not analyze patients separately by dialysis modality. Since hyperkalemia is probably less common in peritoneal dialysis, the prevalence in hemodialysis may be even higher.

In conclusion, we found a high prevalence of hyperkalemia in a large Brazilian chronic dialysis population. A national investigation of risk factors, treatment options, and whether this high prevalence contributes to dialysis mortality is necessary.
